# The Regulation of γ-Aminobutyric Acid on Antioxidative Defense Response of Pacific Oyster upon High-Temperature Stress

**DOI:** 10.3390/antiox14020222

**Published:** 2025-02-15

**Authors:** Ranyang Liu, Lei Gao, Xueshu Zhang, Pingan Ge, Ling Wang, Keli Zhou, Chuanyan Yang, Lingling Wang, Linsheng Song

**Affiliations:** 1 College of Life Sciences, Liaoning Normal University, Dalian 116029, China; liuranyang666@163.com (R.L.); lshsong@dlou.edu.cn (L.S.); 2 Liaoning Key Laboratory of Marine Animal Immunology and Disease Control, Dalian Ocean University, Dalian 116023, China; gaolei@dlou.edu.cn (L.G.); zhangxueshu@dlou.edu.cn (X.Z.); gpa725@163.com (P.G.); wangjieer2022@163.com (L.W.); zhoukeli2024@163.com (K.Z.); yangchuanyan@dlou.edu.cn (C.Y.); 3 Dalian Key Laboratory of Aquatic Animal Disease Prevention and Control, Dalian Ocean University, Dalian 116023, China; 4 Liaoning Key Laboratory of Marine Animal Immunology, Dalian Ocean University, Dalian 116023, China; 5 Southern Marine Science and Engineering Guangdong Laboratory (Zhuhai), Zhuhai 519000, China

**Keywords:** *Crassostrea gigas*, high temperature, antioxidant, GABA, Nrf2, AKT, GSK-3β

## Abstract

Recent studies have found that high temperatures cause oxidative stress and even mass mortality in Pacific oysters (*Crassostrea gigas*). The role of γ-aminobutyric acid (GABA) in improving antioxidative defense in aquatic animals is increasingly of interest. In the present study, the oxidative stress of Pacific oysters to high-temperature stress was examined, and the regulation of GABA on the antioxidative defense was further investigated. Following 6 h of exposure to 28 °C seawater, a significant increase in the mRNA expression levels of nuclear factor-E2-related factor 2 (Nrf2), superoxide dismutase (SOD), and catalase (CAT), as well as the activities of SOD and CAT, was observed in the gill, compared to those at 0 h. An increase of glutamate decarboxylase (GAD), GABA receptor (GABA_A_R-α and GABA_B_R-B) mRNA levels, and GABA contents were also detected after 28 °C exposure compared to those at 0 h. Furthermore, the activities and mRNA expression levels of SOD and CAT were significantly upregulated after GABA treatment, while decreased after either GAD inhibitor or GABA receptor inhibitor treatment under high-temperature stress. Meanwhile, the enhanced effects of GABA on antioxidant enzyme activities were reduced when Nrf2 was inhibited by ML385, accompanied by an increase in MDA content. After high-temperature stress, compared with the GABA treatment group, the activities and mRNA expression levels of SOD and CAT were significantly upregulated by GSK-3β inhibitor treatment. Meanwhile, the elevation of antioxidant enzyme activities by GABA was attenuated by the AKT inhibitor treatment. Collectively, GABA first activated GABA receptors under high-temperature stress and then increased the activities of SOD and CAT and reduced MDA content by AKT/GSK-3β and Nrf2 pathways to protect the oysters against oxidative damage upon stress. The present results offer new insights for understanding the regulation mechanisms of antioxidative defense by the neuroendocrine system in molluscs.

## 1. Introduction

Elevated ocean temperature disrupts reduction/oxidation homeostasis, leading to oxidative damage that can affect the growth and survival of aquatic animals, especially immobile sessile mollusc [1,2,3]. The accumulation of reactive oxygen species (ROS) caused by high-temperature stress has been shown to damage cell membranes and proteins and produce biomarkers of oxidative stress damage such as malondialdehyde (MDA) [4]. Aquatic animals have evolved complex enzymatic and non-enzymatic antioxidative defenses that are effective in blocking harmful effects of ROS to cope with high-temperature stress [5]. Recent evidence suggests that enzymatic antioxidative defense represented by superoxide dismutase (SOD) and catalase (CAT) is finely regulated by neurotransmitters and hormones [6,7].

Nuclear factor-E2-related factor 2 (Nrf2) is a nuclear transcription factor that controls the expression and coordinated induction of a battery of defensive genes encoding antioxidant enzymes and is essential for cellular resistance to oxidative damage and cell survival. The role of Nrf2 in activating antioxidative defense was identified in a variety of molluscs, such as *Crassostrea gigas*, *Mytilus coruscus*, *Ruditapes philippinarum*, and *Cristaria plicata* [8,9,10,11]. In response to high-temperature stress, Nrf2 is stabilized and translocated to the nucleus, where it activates the transcription of SOD and CAT [12]. In molluscs, Nrf2, SOD, and CAT have been identified to evaluate the antioxidative defense capacity under high-temperature stress [13,14]. The antioxidative defense process involves the activation and regulation of Nrf2 in vertebrates. Proteasomal degradation mediated by Kelch-like ECH-associated protein 1 (Keap1) is considered to be the classical regulatory mechanism of Nrf2, which keeps lower Nrf2 levels in normal physiological conditions and increases in response to stress [15,16]. Meanwhile, recent studies have revealed that the glycogen synthase kinase (GSK-3β), a Keap1-independent mechanism, phosphorylates Nrf2, leading to its nuclear exclusion and degradation [17,18]. The regulatory effect of Keap1 on Nrf2 has also been demonstrated in molluscs [19,20]. Nevertheless, research on the Nrf2 regulatory mechanism by GSK-3β in mollusc is currently limited.

Recent research has unveiled the novel role of γ-aminobutyric acid (GABA), a four-carbon nonessential amino acid, in combating oxidative stress. In rat kidney, the administration of GABA attenuated oxidative stress induced by nephrectomy through an increase of SOD and CAT [21]. The activities of SOD and CAT in chicken serum and liver were enhanced by dietary GABA supplementation [22]. In the liver, GABA effectively mitigated oxidative damage triggered by acute liver failure by upregulating SOD protein expression level [23]. These results highlight the role of GABA in enhancing antioxidative defense in vertebrates. GABA is synthesized from glutamate by glutamate decarboxylase (GAD) and acts by binding to GABA_A_ and GABA_B_ receptors. GABA_A_ receptors are ligand-gated chloride channels influenced by various drugs, while GABA_B_ receptors, linked to potassium or calcium channels, function via a GTP-binding protein [24]. In human M1 macrophages, the activation of the GABA receptor induces cytoplasmic accumulation and nuclear translocation of Nrf2 [25]. Meanwhile, in rat insulin-producing cells, GABA exerts its antioxidative defense effect by regulating GSK-3β and Nrf2 [26]. Therefore, based on the inhibitory effect of GSK-3β on Nrf2, the serine–threonine kinase (Akt) is thought to be a possible bridge between GABA and Nrf2. On the one hand, since GSK-3β is inhibited by AKT, activation of the Akt signaling pathway has been found to prevent methylglyoxal- and rotenone-induced oxidative stress through upregulating Nrf2 in neuroblastoma [27,28]. On the other hand, GABA activates Akt through both GABA_A_ receptor and GABA_B_ receptor in human islets [29]. These results highlight the mechanism of GABA in enhancing antioxidative defense in vertebrates. Recent evidence suggests that GABA enhances the antioxidative defense capacity of aquatic invertebrates, enabling them to better adapt to drastic environmental changes. For instance, GABA enhances antioxidative defenses represented by SOD in *Litopenaeus vannamei*, *Eriocheir sinensis*, and *Sepia pharaonics* [30,31,32]. Despite these findings, our understanding of how GABA regulates antioxidative defense in aquatic invertebrates remains a concern.

The Pacific oyster (*C. gigas*), an intertidal sessile mollusc, holds significant aquaculture economic and ocean ecological importance [33]. Amplified by global warming, elevated ocean temperature has triggered oxidative damage and even mortality events during summer, severely impeding the sustainable development of oyster aquaculture [34]. The understanding of physiological responses, particularly defense mechanisms against oxidative damage, to high-temperature stress is key to improving the tolerance, resilience, and sustainability of oyster aquaculture. The antioxidative defense dependent on Nrf2, SOD, and CAT was confirmed to be the basis for the adaptation of oysters to environmental stress [35,36]. Furthermore, the neurotransmitters and hormones play an important role in adaptation to harsh environmental stress by regulating a variety of physiological activities in oysters [37]. In this study, the mRNA expressions of *Cg*GABA_A_R-α and *Cg*GABA_B_R-B, *Cg*Nrf2, *Cg*SOD, *Cg*CAT, and *Cg*GAD; the activities of SOD and CAT; and the contents of MDA and GABA were investigated under high-temperature stress in oysters with the following main objectives: (1) examine activation of antioxidative defense and the GABA system after high-temperature stress; (2) determine the effect of GABA on antioxidant enzymes; and (3) elucidate the mechanisms of GABA in regulating antioxidative defense in oysters. The findings aim to provide new insights into the function of the neuroendocrine system in response to high-temperature stress in oysters.

## 2. Materials and Methods

### 2.1. Animal and Exposure Experiment

Pacific oysters were collected from a local farm in Dalian, Liaoning Province, China. The oysters of similar size were selected, with an average shell length of 130 ± 10 mm and a soft tissue weight of 22 ± 3 g. These oysters were cultured in aerated seawater at a temperature of 18 ± 1 °C and fed with algae powder once daily for two weeks. All the experiments were performed following the animal ethics guidelines approved by the Ethics Committee of Dalian Ocean University.

**Figure 1:**
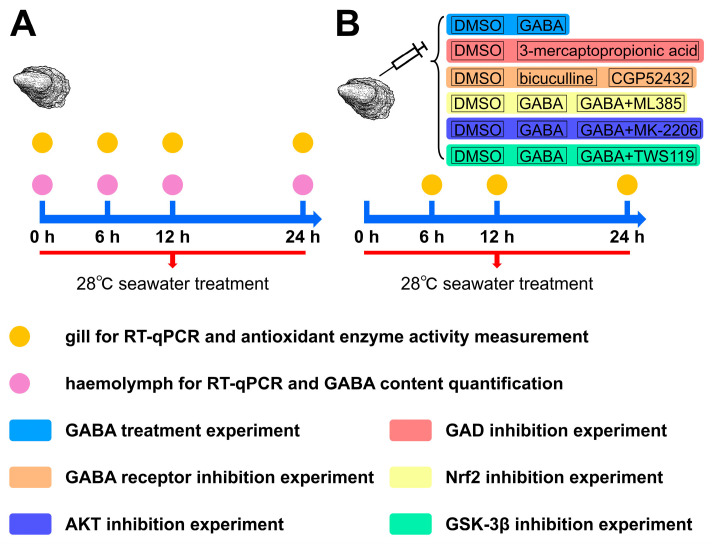
Schematic representation of the experimental design. High-temperature exposure experiment (**A**); GABA functional validation experiment (**B**).

The overview of experimental design is shown in Figure 1. In the high-temperature exposure experiment, 36 oysters were transferred from 18 °C seawater to a 100 L tank with aerated seawater maintained at 28 ± 1 °C. Gill and haemolymph were collected from nine oysters at 0, 6, 12, and 24 h post-exposure. To ensure sufficient materials for analysis of all indices, gill and haemolymph from three oysters were pooled into one sample, and there were three biological replicates for each sampling at different time points. The 0 h indicates that oysters are not yet exposed to high-temperature stress at this time. Haemolymph was centrifuged at 800× *g* at 4 °C for 10 min to separate haemocytes and supernatant. Haemolymph supernatant was preserved at −80 °C for quantification of GABA contents. Haemocytes and half of the gill samples were either treated with 1 mL of TRIzol (Thermo Fisher Scientific, Waltham, MA, USA) and stored at −80 °C for RNA extraction, and the other half of the gill samples were directly frozen at −80 °C for the determination of antioxidant enzyme activity and MDA content. Three parallel biological replicates were used in each measurement.

The GABA functional validation experiment consisted of six parts: the GABA treatment experiment, the GAD inhibition experiment, the GABA receptor inhibition experiment, the Nrf2 inhibition experiment, the AKT inhibition experiment, and the GSK-3β inhibition experiment. In each experiment, each group consisted of 27 randomly selected oysters, and different groups of oysters were placed in their own separate tanks. The oysters in the DMSO group received an injection of 100 μL of sterilized seawater containing 1% DMSO into the adductor muscles. Oysters in other groups were injected into the adductor muscle with 100 μL of respective drugs (GABA, 20 mg/kg; 3-mercaptopropionic acid, 40 mg/kg; bicuculline, 5 mg/kg; CGP52432, 5 mg/kg; ML385, 10 mg/kg; MK-2206, 10 mg/kg; TWS119, 10 mg/kg; dissolved in 1% DMSO). 3-mercaptopropionic acid, bicuculline, CGP52432, ML385, MK-2206, and TWS119 were selected as inhibitors of GAD, GABA_A_ receptor, GABA_B_ receptor, AKT, and GSK-3β, respectively, according to the previous description [38,39,40,41]. After injection, the different groups of oysters were placed into their respective 100 L tanks with 28 ± 1 °C seawater, respectively. Nine oysters were sampled at 6, 12, and 24 h post-exposure. The gills from three oysters were mixed together as one replicate, and there were three parallel replicates at each time point. Half of the gill samples were either treated with 1 mL of TRIzol and stored at −80 °C for RNA extraction, and the other half of the samples were directly frozen at −80 °C for the determination of antioxidant enzyme activity and MDA content. Three parallel biological replicates were used in each measurement.

### 2.2. RNA Extraction and cDNA Synthesis

Total RNA was extracted from the collected tissues using Trizol (Invitrogen) reagent, following the standard protocol [42]. cDNA was synthesized using TransScript^®^ One-Step gDNA Removal and cDNA Synthesis SuperMix Kit (TransGen, Beijing, China) in accordance with the instructions of the manufacturer. The resulting cDNA was diluted to 1:20 and stored at −80 °C for subsequent analysis.

### 2.3. RT-qPCR Analysis for mRNA Expression of Genes

The RT-qPCR was performed using the TransStart^®^ Tip Green qPCR SuperMix (+Dye II, TransGen Biotech, Beijing, China) on an ABI 7500 Real-Time Detection System (Applied Biosystems, Bedford, MA, USA). The elongation factor (*Cg*EF, NM_001305313) of Pacific oyster was used as the reference gene [43]. The relative expression levels of *Cg*Nrf2, *Cg*SOD, *Cg*CAT, *Cg*GABA_A_R-α, *Cg*GABA_B_R-B, and *Cg*GAD were analyzed using the comparative Ct method (2^−ΔΔCt^). The primers used in this study are listed in Table 1.

### 2.4. Quantification of GABA Content

The content of GABA in haemolymph supernatant was quantified by GABA ELISA Kit (ml770288, Mlbio, Shanghai, China), based on a double antibody sandwich method [44].

### 2.5. Measurement of Oxidative Stress Indexes

The gill tissues were mixed with saline solution to create a 10% tissue homogenate for enzyme activity measurement. SOD activity, CAT activity, and MDA content were measured using the superoxide dismutase assay kit (A001-3-2, WBT-1 method), the catalase assay kit (A007-1-1, ammonium molybdate method), and the malondialdehyde assay kit (A-003-1-2), respectively, according to the instruction manual (Jian Cheng Bioengineering Institute, Nanjing, China) and previous description [45].

### 2.6. Statistical Analysis

All statistical analyses were carried out using Statistical Package for Social Sciences (SPSS) version 20.0. The normality of the data distributions was checked using a Shapiro–Wilk’s test. The data in the high-temperature exposure experiment were subjected to one-way analysis of variance (one-way ANOVA), followed by a post hoc multiple-comparison (Tukey’s) test. Differences were considered statistically significant at *p* < 0.05. Significant differences between groups are indicated by different letters (*p* < 0.05). Student’s *t*-test was used to analyze significant differences between the different groups in the GABA functional validation experiment. The Mann–Whitney test was used for non-normally distributed data. Asterisks indicated significant differences (* *p* < 0.05, ** *p* < 0.01, and *** *p* < 0.001).

## 3. Results

### 3.1. The SOD Activity, CAT Activity, MDA Content, and the mRNA Expression Levels of CgSOD, CgCAT, and CgNrf2 in Gill After High-Temperature Stress

**Figure 2:**
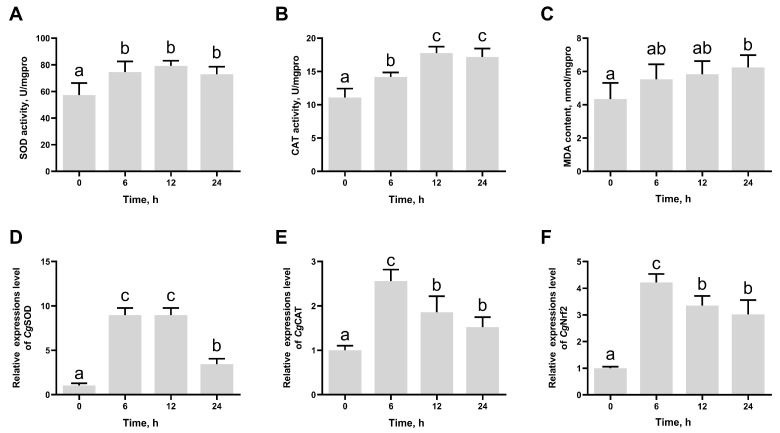
The activities of SOD (**A**) and CAT (**B**); MDA content (**C**); mRNA expression levels of *Cg*SOD (**D**), *Cg*CAT (**E**), and *Cg*Nrf2 (**F**) in gill at 0, 6, 12, and 24 h post high-temperature stress. The bars represent the mean ± SD (*n* = 3). Significant differences between groups are indicated by different letters (*p* < 0.05).

The biochemical indexes, CAT, SOD, and MDA, were used to evaluate the effects of high-temperature exposure on oxidative stress in Pacific oysters. SOD activity and CAT activity increased significantly from 6 h after high-temperature stress and reached the peak at 12 h, which were 1.38-fold (*p* = 0.020205) and 1.60-fold (*p* = 0.000309) of those at 0 h (Figure 2A,B). The MDA content was significantly higher at 24 h (1.44-fold, *p* = 0.025884) after high-temperature stress compared to that at 0 h (Figure 2C). The mRNA expression levels of *Cg*SOD and *Cg*CAT exhibited a trend of initial increase followed by a decrease, reaching the peak at 6 h, which were 8.76-fold (*p* = 0.000002) and 2.55-fold (*p* = 0.000315) of those at 0 h (Figure 2D,E). The mRNA expression levels of *Cg*Nrf2 increased significantly from 6 h to 24 h after high-temperature stress and reached the peak at 6 h, which was 4.22-fold (*p* = 0.000032) of that at 0 h (Figure 2F).

### 3.2. The Change in CgGAD, CgGABA_A_R-α, and CgGABA_B_R-B Expression and GABA Content After High-Temperature Stress

**Figure 3:**
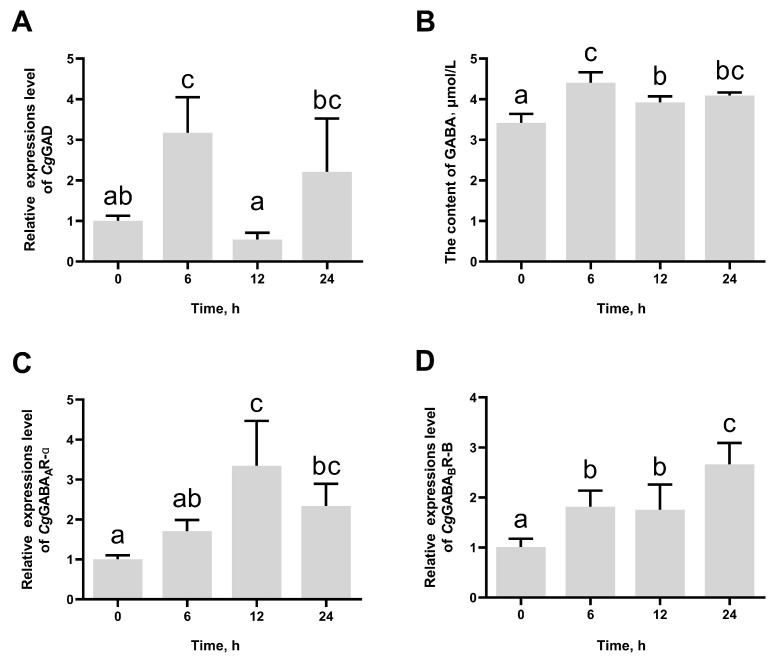
The mRNA expression levels of *Cg*GAD (**A**) in haemocytes, the GABA content (**B**) in haemolymph, and mRNA expression levels of *Cg*GABA_A_R-α (**C**) and *Cg*GABA_B_R-B (**D**) in gills at 0, 6, 12, and 24 h post high-temperature stress. The bars represent the mean ± SD (*n* = 3). Significant differences between groups are indicated by different letters (*p* < 0.05).

The mRNA expression levels of *Cg*GAD in haemocytes increased quickly and peaked at 6 h (3.16-fold of that at 0 h, *p* = 0.041667) after high-temperature stress and then reversed to the original level at 12 h and 24 h (Figure 3A). In addition, GABA contents in haemolymph supernatant significantly increased at 6 h (4.41 μmol/L, *p* = 0.000940), 12 h (3.92 μmol/L, *p* = 0.044654), and 24 h (4.09 μmol/L, *p* = 0.010419) after high-temperature stress compared to that at 0 h (3.41 μmol/L) (Figure 3B). The mRNA expression levels of *Cg*GABA_A_R-α and *Cg*GABA_B_R-B increased significantly after high-temperature stress and reached the peak at 12 h and 24 h, respectively, which were 3.35-fold (*p* = 0.009189) and 2.67-fold (*p* = 0.002813) of those at 0 h (Figure 3C,D).

### 3.3. The SOD Activity, CAT Activity, MDA Content, and the mRNA Expression Levels of CgGABA_A_R-α, CgGABA_B_R-B, CgSOD, CgCAT, and CgNrf2 in Gills After GABA Treatment

**Figure 4:**
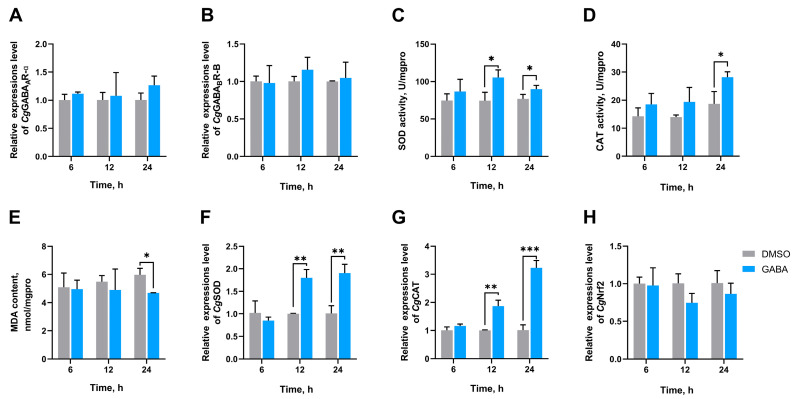
The mRNA expression levels of *Cg*GABA_A_R-α (**A**) and *Cg*GABA_B_R-B (**B**; activities of SOD (**C**) and CAT (**D**); MDA content (**E**); and mRNA expression levels of *Cg*SOD (**F**), *Cg*CAT (**G**), and *Cg*Nrf2 (**H**) in gills of GABA-injected oysters at 6, 12, and 24 h post high-temperature stress. The bars represent the mean ± SD (*n* = 3). Asterisks indicate significant differences (* *p* < 0.05, ** *p* < 0.01, and *** *p* < 0.001).

To investigate the effect of GABA on antioxidative defense, oysters were exposed to high temperatures after the injection with GABA. The SOD activity of the GABA group significantly increased at 12 h (1.41-fold, *p* = 0.023780) and 24 h (1.20-fold, *p* = 0.044724) after high-temperature stress compared to that in the DMSO group (Figure 4C). The CAT activity was significantly increased at 24 h after injection of GABA, which was 1.51-fold (*p* = 0.026673) that of the DMSO group (Figure 4D). The MDA content in the GABA group was significantly reduced to 0.79-fold (*p* = 0.040732) of the DMSO group at 24 h (Figure 4E). *Cg*SOD expression levels were significantly increased at 12 h and 24 h after the injection of GABA, which were 1.80-fold (*p* = 0.001602) and 1.89-fold (*p* = 0.003936) of that in DMSO group, respectively (Figure 4F). *Cg*CAT expression levels increased to 1.87-fold (*p* = 0.002010) and 3.19-fold (*p* = 0.000280) at 12 h and 24 h, respectively (Figure 4G). In addition, no significant differences in *Cg*GABA_A_R-α, *Cg*GABA_B_R-B, and *Cg*Nrf2 mRNA expression levels were observed in different groups at all time points (Figure 4A,B,H).

### 3.4. The GABA Content in Haemolymph Supernatant, SOD Activity, CAT Activity, MDA Content, and the mRNA Expression Levels of CgSOD, CgCAT, and CgNrf2 in Gills After GAD Inhibition

**Figure 5:**
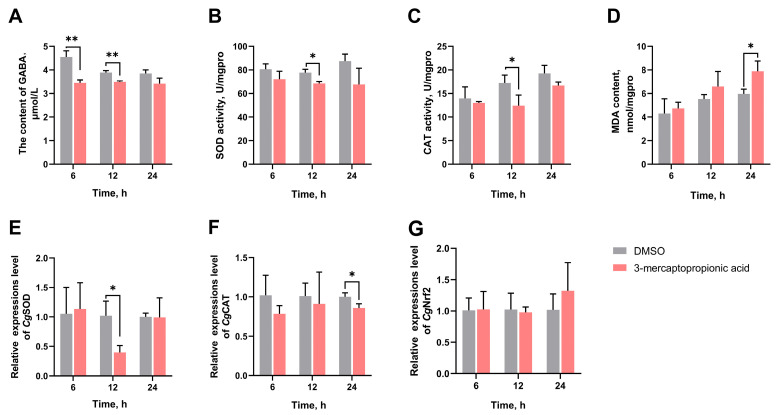
The GABA content (**A**) in haemolymph supernatant, activities of SOD (**B**) and CAT (**C**), MDA content (**D**), mRNA expression levels of *Cg*SOD (**E**), *Cg*CAT (**F**), and *Cg*Nrf2 (**G**) in gills of GAD inhibitor-injected oysters at 6, 12, and 24 h post high-temperature stress. The 3-mercaptopropionic acid was selected as an inhibitor of GAD. The bars represent the mean ± SD (*n* = 3). Asterisks indicate significant differences (* *p* < 0.05, ** *p* < 0.01).

The 3-mercaptopropionic acid was injected into oysters to inhibit the GABA synthesis induced by high-temperature stress. The GABA content of the 3-mercaptopropionic acid group was significantly reduced to 0.76-fold (*p* = 0.003138) and 0.89-fold (*p* = 0.001798) of the DMSO group at 6 h and 24 h, respectively (Figure 5A). The SOD activity and CAT activity of the 3-mercaptopropionic acid group were significantly reduced to 0.88-fold (*p* = 0.014590) and 0.72-fold (*p* = 0.042864) of the DMSO group at 12 h, respectively (Figure 5B,C). The MDA content was significantly increased to 1.32-fold (*p* = 0.024520) of the DMSO group at 24 h (Figure 5D). After 3-mercaptopropionic acid treatment, the mRNA expression levels of *Cg*SOD and *Cg*CAT significantly reduced to 0.39-fold (*p* = 0.017598) and 0.86-fold (*p* = 0.028408) of the DMSO group at 12 h and 24 h, respectively (Figure 5E,F). In addition, no significant difference was observed in *Cg*Nrf2 mRNA expression level in the different groups at all time points (Figure 5G).

### 3.5. The SOD Activity, CAT Activity, MDA Content, and the mRNA Expression Levels of CgSOD, CgCAT, and CgNrf2 in Gills After Treatment with GABA Receptor Inhibitors

**Figure 6:**
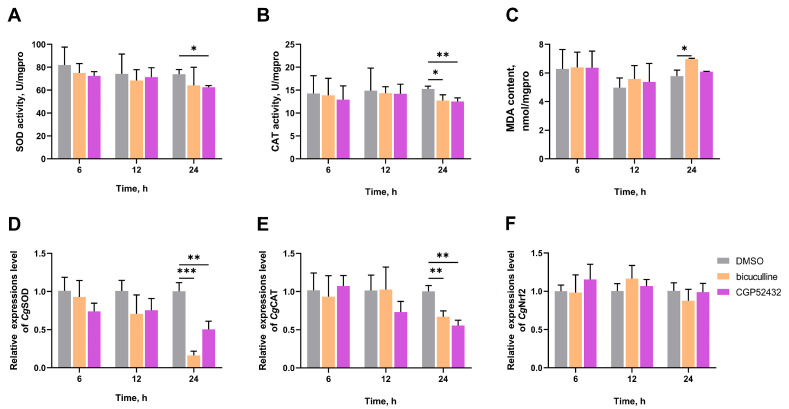
The activities of SOD (**A**) and CAT (**B**); MDA content (**C**); and mRNA expression levels of *Cg*SOD (**D**), *Cg*CAT (**E**), and *Cg*Nrf2 (**F**) in gills of GABA receptor inhibitor-injected oysters at 6, 12, and 24 h after high-temperature stress. The bicuculline and CGP52432 were selected as inhibitors of GABA_A_ and GABA_B_ receptors. The bars represent the mean ± SD (*n* = 3). Asterisks indicated significant differences (* *p* < 0.05, ** *p* < 0.01, and *** *p* < 0.001).

To explore the pathway by which GABA promotes antioxidative defense, bicuculline and CGP52432 are used to block GABA receptors. The SOD activity of the CGP52432 group was significantly reduced to 0.85-fold (*p* = 0.029055) of the DMSO group at 24 h (Figure 6A). The CAT activities of the bicuculline and CGP52432 groups were significantly reduced to 0.83-fold (*p* = 0.033506) and 0.82-fold (*p* = 0.008167) of the DMSO group at 24 h, respectively (Figure 6B). The MDA content of the bicuculline group was significantly increased to 1.21-fold (*p* = 0.036363) of the DMSO group at 24 h (Figure 6C). Following high-temperature stress, the mRNA expression levels of *Cg*SOD and *Cg*CAT were significantly altered by injections of bicuculline and CGP52432. *Cg*SOD expression levels reduced to 0.50-fold (bicuculline group, *p* = 0.000300) and 0.16-fold (CGP52432 group, *p* = 0.004967) of the DMSO group at 24 h, respectively (Figure 6D). Similar trends were also seen in the mRNA expression levels of *Cg*CAT. *Cg*CAT expression levels reduced to 0.67-fold (bicuculline group, *p* = 0.006387) and 0.56-fold (CGP52432 group, *p* = 0.001715) of the DMSO group at 24 h, respectively (Figure 6E). In addition, no significant difference was observed in *Cg*Nrf2 mRNA expression level of different groups at all time points (Figure 6F).

### 3.6. The SOD Activity, CAT Activity, and MDA Content in Gills After Nrf2 Inhibition

**Figure 7:**
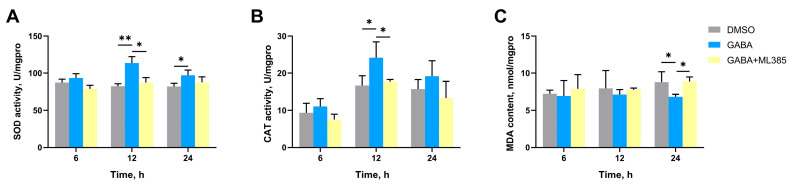
The activities of SOD (**A**) and CAT (**B**) and MDA content (**C**) in gills of Nrf2 inhibitor-injected oysters at 6, 12, and 24 h after high-temperature stress. ML385 was selected as an inhibitor of Nrf2. The bars represent the mean ± SD (*n* = 3). Asterisks indicated significant differences (* *p* < 0.05, ** *p* < 0.01).

The SOD activity and CAT activity of GABA group significantly increased to 1.38-fold (*p* = 0.004327) and 1.22-fold (*p* = 0.043694) of DMSO group at 12 h, respectively (Figure 7A,B). The MDA content of the GABA group was significantly reduced to 0.77-fold (*p* = 0.035159) of the DMSO group at 24 h (Figure 7C). In the GABA + ML385 group, no significant difference was observed in SOD activity, CAT activity, and MDA content of the DMSO group at the same time points (Figure 7A–C).

### 3.7. The SOD Activity, CAT Activity, MDA Content, and the mRNA Expression Levels of CgNrf2, CgSOD, and CgCAT in Gills After AKT Inhibition

**Figure 8:**
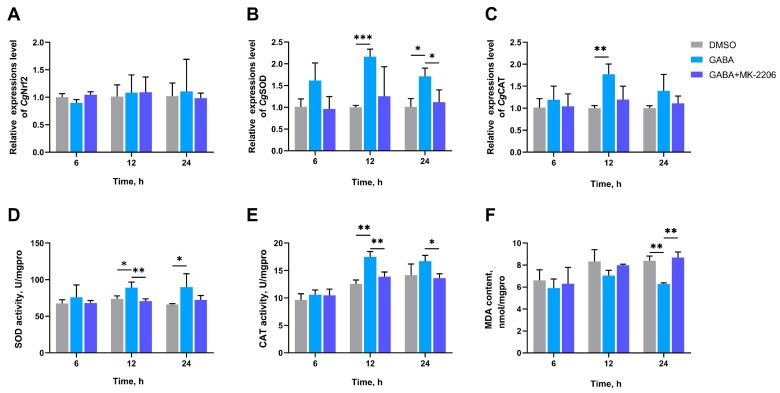
The mRNA expression levels of *Cg*Nrf2 (**A**), *Cg*SOD (**B**), and *Cg*CAT (**C**); activities of SOD (**D**) and CAT (**E**); and MDA content (**F**) in gills of AKT inhibitor-injected oysters at 6, 12, and 24 h after high-temperature stress. MK-2206 was selected as inhibitors of AKT. The bars represent the mean ± SD (*n* = 3). Asterisks indicated significant differences (* *p* < 0.05, ** *p* < 0.01, and *** *p* < 0.001).

To demonstrate the role of GSK-3β in GABA promoting antioxidative defense, MK-2206 was used to reduce the inhibition of GSK-3β by AKT. The mRNA expression levels of *Cg*SOD and *Cg*CAT, SOD activity, and CAT activity in the GABA group significantly increased to 2.16-fold (*p* = 0.000340), 1.77-fold (*p* = 0.004861), 1.21-fold (*p* = 0.036493), and 1.39-fold (*p* = 0.002123) of the DMSO group at 12 h, respectively (Figure 8B–E). The MDA content of the GABA group was significantly reduced to 0.75-fold (*p* = 0.001096) of the DMSO group at 24 h (Figure 8F). In the GABA + MK-2206 group, no significant difference was observed in the mRNA expression of *Cg*Nrf2, *Cg*SOD, and *Cg*CAT, and SOD activity and CAT activity compared with the DMSO group at the same time points (Figure 8A–F).

### 3.8. The SOD Activity, CAT Activity, MDA Content, and the mRNA Expression Levels of CgNrf2, CgSOD, and CgCAT in Gills After GSK-3β Inhibition

**Figure 9:**
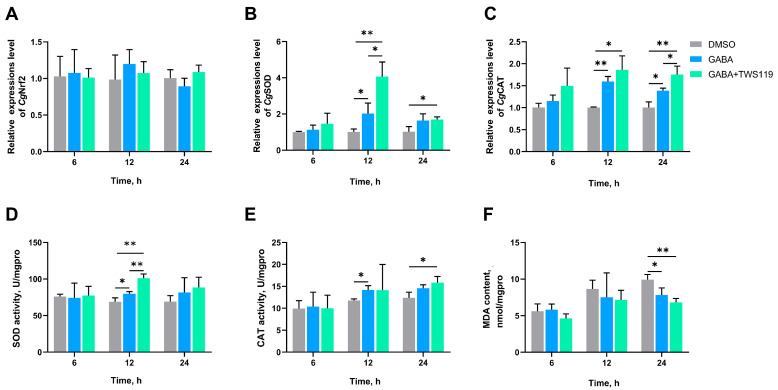
The mRNA expression levels of *Cg*Nrf2 (**A**), *Cg*SOD (**B**), and *Cg*CAT (**C**); activities of SOD (**D**) and CAT (**E**); and MDA content (**F**) in gills of GSK-3β inhibitor-injected oysters at 6, 12, and 24 h after high-temperature stress. TWS119 was selected as inhibitors of GSK-3β. The bars represent the mean ± SD (*n* = 3). Asterisks indicated significant differences (* *p* < 0.05 and ** *p* < 0.01).

In the GSK-3β inhibition experiment, no significant difference was observed in *Cg*Nrf2 mRNA expression levels of different groups at all time points (Figure 9A). In the GABA + TWS119 group, the mRNA expression levels of *Cg*SOD significantly increased to 4.03-fold (*p* = 0.003005) and 2.00-fold (*p* = 0.023865) of these in DMSO group and GABA group at 12 h, respectively (Figure 9B). *Cg*CAT expression levels of GABA + TWS119 group increased to 1.74-fold (*p* = 0.004655) and 1.28-fold (*p* = 0.032556) of these in the DMSO group and the GABA group at 24 h, respectively (Figure 9C). In the GABA + TWS119 group, the SOD activities significantly increased to 1.47-fold (*p* = 0.002013) and 1.27-fold (*p* = 0.004721) of these in the DMSO group and the GABA group at 12 h, respectively (Figure 9D). The CAT activities of the GABA group and the GABA + TWS119 group were significantly increased to 1.20-fold (at 12 h, *p* = 0.017949) and 1.28-fold (at 24 h, *p* = 0.034407) of the DMSO group, respectively (Figure 9E). The MDA contents of the GABA group and the GABA + TWS119 group were significantly reduced to 0.79-fold (*p* = 0.037282) and 0.69-fold (*p* = 0.003640) of the DMSO group at 24 h, respectively (Figure 9F).

## 4. Discussion

Oxidative stress caused by high-temperature stress can lead to oxidative damage and threaten the survival of aquatic animals [46]. Global ocean warming is escalating physiological stress on aquaculture species, compelling the need for a deeper understanding of their stress response and resistance mechanisms. In aquatic animals, increasing attention has been paid to the role of the neuroendocrine system in the regulation of stress response [47]. This study explores the role of GABA in mitigating oxidative damage by examining changes in GABA and MDA contents, antioxidant enzyme activities, and mRNA expression of GABA synthesis and antioxidative defense genes in oysters under high-temperature stress. Our results aim to investigate the regulation mechanisms of the antioxidative defense system by GABA, thereby advancing our knowledge of the role of the neuroendocrine system in bivalves coping with environmental stress.

High temperature induces MDA formation from polyunsaturated fatty acids in membrane lipids by increasing electron leakage from the mitochondrial electron transport chain and the level of ROS. In aquaculture animals, MDA has been widely studied as an indicator of oxidative damage [48]. The coasts of Liaodong Peninsula and Shandong Peninsula are the dominant areas of Pacific oyster farming in north China, where the highest seawater temperature in summer is around 25 °C and 28 °C, respectively [49]. Meanwhile, in a previous study, MDA content of Pacific oysters did not increase significantly below 26 °C, but increased rapidly at 28 °C and 30 °C [43]. Therefore, 28 °C was selected as the condition of high-temperature stress treatment in this study. Gill tissue is essential for respiration and filter feeding in Pacific oyster and is highly sensitive to high-temperature stress [50]. In the present study, 24 h of 28 °C treatment resulted in a significant rise in MDA content in the gills, suggesting that oysters exhibited oxidative damage. This was consistent with the findings of some previous studies in the liver of *Cyprinus carpio* and the gills of *C. gigas* [43,51]. Oxidative stress usually activates antioxidative defense, in which SOD and CAT, as important antioxidant enzymes, are used to indicate the response of aquatic animals to high-temperature stress [14]. In this study, the 28 °C treatment resulted in a significant rise in SOD activity and CAT activity, and the mRNA expression levels of *Cg*SOD and *Cg*CAT in the gills, suggesting that the antioxidative defense of oysters has been activated. The changes of antioxidant enzyme activity in this study showed a trend of first increasing and then decreasing, which was similar to the results of previous experiments under 35 °C and 37 °C exposure [52,53]. There are two different views on this trend regarding the previous research in fish, one view is that the decrease in antioxidant enzyme activity indicates that the organism has adapted to the stress, while the other view is that the decrease reflects the collapse of the antioxidative defense system caused by the stress [54,55]. However, for oysters, more results are still needed to clarify the significance of the observed decrease in antioxidant enzyme activity.

GABA is found to be able to respond to environmental stress and play a role in maintaining reduction/oxidation homeostasis. Environmental stressors trigger the accumulation of GABA in aquatic animals. This was evidenced by significant increases in GABA levels in turtle and crucian carp brains exposed to the anoxia stress [56,57]. In Pacific oysters, the GAD was found to show the highest expression level in haemocytes [58]. Thus, GAD in haemocytes and GABA in haemolymph supernatant were used to indicate the effects of high-temperature stress in the present study, and a substantial rise in *Cg*GAD mRNA expression and GABA content was observed at 6 h after high-temperature exposure, underscoring a correlation between high-temperature stress and GABA synthesis in oysters. The role of GABA in bolstering stress resilience is corroborated through investigations on *S. pharaonis*, where GABA has been shown to reist ammonia toxicity stress by increasing SOD activity and CAT activity [59]. The role of GABA in bolstering antioxidative defense is further supported in the present study, where GABA treatment has been shown to reduce MDA content and increase the SOD activity, CAT activity, and the mRNA expression levels of *Cg*SOD and *Cg*CAT in gill after high-temperature stress. This was consistent with the findings of some previous studies in vertebrates [31]. Meanwhile, inhibition of GABA synthesis by GAD inhibitor reduced the SOD activity and CAT activity. These observations suggest that the GABA accumulation caused by high-temperature stress plays a crucial role in promoting antioxidative defense, thereby reducing oxidative damage in oysters during stress. Although similar studies in mollusc are limited, GABA has been used as a biological indicator to evaluate pollution stress in *T. granosa* and hypoxia stress in *Mytilus edulis* and *C. gigas* [60,61]. These studies confirm the important role of GABA in mollusc response to environmental stress from another perspective.

**Figure 10:**
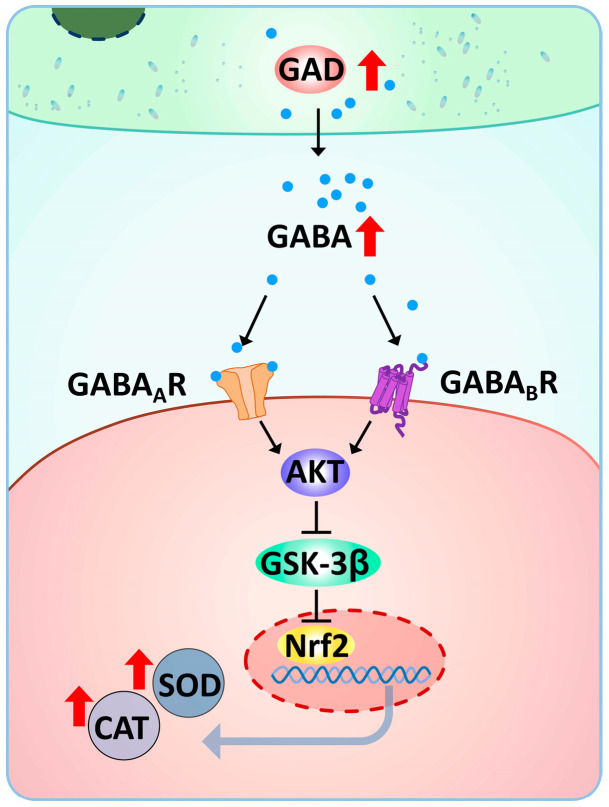
GABA is involved in the antioxidative defense of oysters upon high-temperature stress. GABA activated GABA_A_ and GABA_B_ receptors and then induced the Nrf2 regulation via the AKT/GSK-3β pathway, along with the increasing SOD and CAT activities, to protect from oxidative damage in the response to high-temperature stress.

As chemical mediators of intercellular communication, GABA usually transmits information and regulates cellular activities by activating its specific receptors [62]. Activation of the GABA receptor plays a pivotal role in cellular defense mechanisms against stress [25,63]. GABA receptors have been identified and validated for binding GABA in molluscs [64,65,66]. In the present study, the mRNA expression levels of GABA receptors were increased by high-temperature stress but not by additional GABA treatment. These results suggest that GABA receptors are involved in GABA functioning in response to high-temperature stress, but that GABA does not directly regulate the expression of GABA receptors. The bicuculline and CGP52432 act as specific competitive antagonists of GABA_A_ and GABA_B_ receptors, respectively, competitively inhibiting the binding of GABA to these receptors [67]. In the present study, the treatment of bicuculline and CGP52432 resulted in the reduced expression of *Cg*SOD and *Cg*CAT, thereby indicating the participation of both receptors in the regulation of antioxidative defense by GABA. This result is similar to the observation in *T. granosa*, suggesting that GABA_A_ and GABA_B_ receptors may induce similar downstream genes in the response to stressors [38]. Nrf2 was confirmed to be the target of GABA receptor activation in vertebrates. In human M1 macrophages, GABA_A_ receptor activation was found to induce nuclear translocation of Nrf2 [25]. The findings in ileal tissues of mice demonstrate that activation of the GABA_B_ receptor leads to the enhancement of Nrf2 signaling [68]. Moreover, Nrf2 plays a crucial role in defending against oxidative stress caused by high-temperature stress and enhancing antioxidant responses [69]. In Pacific oysters, Nrf2 has been identified as a function in the transcriptional induction of antioxidant enzyme [8,19,43]. In this study, the expression level of Nrf2 mRNA in oyster gills increased significantly after high-temperature stress, while remaining at the original level after GABA treatment. These results differed from previous studies in the liver of largemouth bass under flow velocity stress and crucian carp under ammonia stress, where GABA led to increased mRNA expression levels of Nrf2 [70,71]. This finding may indicate differences in GABA regulation of different stress responses or different oxidative stress levels. Furthermore, the enhanced effects of GABA on antioxidant enzyme activities were reduced when Nrf2 was inhibited by ML385. These results suggest that GABA enhanced antioxidant enzyme activities in a Nrf2-dependent manner but did not regulate Nrf2 transcription in oyster gill. Nrf2 acts as a transcription factor to regulate various downstream antioxidant enzymes through nuclear translocation and subsequent binding to antioxidant response elements in the promoter region [72]. GSK-3β is thought to be a key molecule as a bridge between GABA and Nrf2 [26]. It has been demonstrated that AKT, the upstream regulator of GSK-3β, is activated by GABA and inhibits GSK-3β activity [29,73,74], while GSK-3β causes nuclear exclusion and degradation of Nrf2 [75,76]. In previous studies, the homologs of AKT and GSK-3β have been identified from the Pacific oysters [77,78]. In the present study, the elevation of antioxidant enzyme activities by GABA was potentiated by the GSK-3β inhibitor. Meanwhile, the treatment of the AKT inhibitor led to the opposite effect. These results suggest that GABA plays a role in enhancing the antioxidative defense of oyster response to high-temperature stress possibly through the AKT/GSK3β/Nrf2 pathway. Collectively, the transcripts of *Cg*GAD in haemocytes and GABA content in haemolymph supernatant increased by high-temperature stress. Upon high-temperature stress, the accumulated GABA activated GABA_A_ and GABA_B_ receptors and then induced the Nrf2 regulation via the AKT/GSK-3β pathway, with the increasing SOD and CAT activities and reducing MDA contents to protect from oxidative damage (Figure 10). GABA as a food additive has been found to significantly increase SOD and CAT activities and enhance stress resistance in aquaculture animals during exposure to environmental stress [59]. Although oyster farming does not rely on artificial feeding, increasing the number of GABA-rich algal species in summer by appropriately altering the nutrient content of the seawater in oyster farming areas may be a potential means to reduce the high temperature-induced mass mortality of Pacific oysters.

## 5. Conclusions

The present study clearly confirmed that the neuroendocrine system is involved in the response of oysters upon high-temperature stress and that GABA regulates antioxidative defense. Specifically, GABA in Pacific oysters accumulated after high-temperature stress and increased SOD and CAT activities through the AKT/GSK-3β/Nrf2 pathway dependent on GABA receptors. Our findings provide novel insights into the response of oysters to high-temperature stress in the context of global warming and clues to develop strategies for the improvement of oyster aquaculture.

## Figures and Tables

**Table 1:** Primers used for RT-qPCR in this study.

Primer Name	Sequence (5′–3′)
*Cg*EF-RT-F	AGTCACCAAGGCTGCACAGAAAG
*Cg*EF-RT-R	TCCGACGTATTTCTTTGCGATGT
*Cg*GAD-RT-F	GCTATGTGCGGATTACCTCTACCAG
*Cg*GAD-RT-R	GATTCGCTAAGTCTTGGGTTGGATA
*Cg*GABA_A_R-α-RT-F	GAGTTCTTTTAGCGGCCGTG
*Cg*GABA_A_R-α-RT-R	TGCAGACGTTCAGGAAGACG
*Cg*GABA_B_R-B-RT-F	CACCTTCATGCTGACGTCCT
*Cg*GABA_B_R-B-RT-R	TCCACCAAAACCGCACCTTT
*Cg*Nrf2-RT-F	ACACAGCCTGTCAGACTTCAC
*Cg*Nrf2-RT-R	CACATCGAACATCTCCTTCCCT
*Cg*SOD-RT-F	TGACAGAAGCGTCCGTTGGC
*Cg*SOD-RT-R	CCGCCTTGAATCTTTCGTTG
*Cg*CAT-RT-F	AACTACTTCGCTGAGGTG
*Cg*CAT-RT-R	GGTCTTGGCTTTGTATGG

## Data Availability

Data will be made available upon request.

## References

[1] Gervais C.R., Huveneers C., Rummer J.L., Brown C. (2021). Population variation in the thermal response to climate change reveals differing sensitivity in a benthic shark. Glob. Chang. Biol..

[2] Schnytzer Y., Simon-Blecher N., Li J., Waldman Ben-Asher H., Salmon-Divon M., Achituv Y., Hughes M.E., Levy O. (2018). Tidal and diel orchestration of behaviour and gene expression in an intertidal mollusc. Sci. Rep..

[3] Masanja F., Yang K., Xu Y., He G., Liu X., Xu X., Xiaoyan J., Xin L., Mkuye R., Deng Y. (2023). Impacts of marine heat extremes on bivalves. Front. Mar. Sci..

[4] Kim M.J., Kim J.A., Lee D.W., Park Y.S., Kim J.H., Choi C.Y. (2023). Oxidative stress and apoptosis in disk abalone (*Haliotis discus hannai*) caused by water temperature and pH changes. Antioxidants.

[5] Birnie-Gauvin K., Costantini D., Cooke S.J., Willmore W.G. (2017). A comparative and evolutionary approach to oxidative stress in fish: A review. Fish Fish..

[6] Chainy G.B.N., Sahoo D.K. (2020). Hormones and oxidative stress: An overview. Free Radic. Res..

[7] Kloet E.R.d., Kloet S.F.d., Kloet C.S.d., Kloet A.D.d. (2019). Top-down and bottom-up control of stress-coping. J. Neuroendocrinol..

[8] Danielli N.M., Trevisan R., Mello D.F., Fischer K., Deconto V.S., da Silva Acosta D., Bianchini A., Bainy A.C.D., Dafre A.L. (2017). Upregulating Nrf2-dependent antioxidant defenses in Pacific oysters *Crassostrea gigas*: Investigating the Nrf2/Keap1 pathway in bivalves. Comp. Biochem. Physiol. Part C Toxicol. Pharmacol..

[9] Qiu L., Chen X., Guo B., Liao Z., Buttino I., Yan X., Qi P. (2023). Unraveling the protective role of Nrf2 in molluscs: Insights into mitochondrial and apoptosis pathways in the defense against Bap-induced oxidative stress. Aquat. Toxicol..

[10] Wang H., Pan L., Si L., Miao J. (2018). The role of Nrf2-Keap1 signaling pathway in the antioxidant defense response induced by PAHs in the calm *Ruditapes philippinarum*. Fish Shellfish Immunol..

[11] Feng M., Hu Y., Yang L., Wu J., Yang G., Jian S., Hu B., Wen C. (2023). GST-Mu of *Cristaria plicata* is regulated by Nrf2/Keap1 pathway in detoxification microcystin and has antioxidant function. Aquat. Toxicol..

[12] Nguyen T., Nioi P., Pickett C.B. (2009). The Nrf2-Antioxidant Response Element Signaling Pathway and Its Activation by Oxidative Stress. J. Biol. Chem..

[13] Abramov T., Suwansaard S., da Silva P.M., Wang T., Dove M., O’Connor W., Parker L., Lovejoy D.A., Cummins S.F., Elizur A. (2022). Teneurin and TCAP phylogeny and physiology: Molecular analysis, immune activity, and transcriptomic analysis of the stress response in the Sydney rock oyster (*Saccostrea glomerata*) hemocytes. Front. Endocrinol..

[14] Rahman M.A., Henderson S., Miller-Ezzy P., Li X.X., Qin J.G. (2019). Immune response to temperature stress in three bivalve species: Pacific oyster *Crassostrea gigas*, Mediterranean mussel *Mytilus galloprovincialis* and mud cockle *Katelysia rhytiphora*. Fish Shellfish Immunol..

[15] Kopacz A., Kloska D., Forman H.J., Jozkowicz A., Grochot-Przeczek A. (2020). Beyond repression of Nrf2: An update on Keap1. Free Radic. Biol. Med..

[16] Glory A., Averill-Bates D.A. (2016). The antioxidant transcription factor Nrf2 contributes to the protective effect of mild thermotolerance (40 °C) against heat shock-induced apoptosis. Free Radic. Biol. Med..

[17] Yu C., Xiao J.H. (2021). The Keap1-Nrf2 System: A Mediator between Oxidative Stress and Aging. Oxid. Med. Cell. Longev..

[18] Rada P., Rojo A.I., Evrard-Todeschi N., Innamorato N.G., Cotte A., Jaworski T., Tobón-Velasco J.C., Devijver H., García-Mayoral M.F., Van Leuven F. (2012). Structural and functional characterization of Nrf2 degradation by the glycogen synthase kinase 3/β-TrCP axis. Mol. Cell. Biol..

[19] Danielli N.M., Trevisan R., Mello D.F., Fischer K., Deconto V.S., Bianchini A., Bainy A.C.D., Dafre A.L. (2017). Contrasting effects of a classic Nrf2 activator, tert-butylhydroquinone, on the glutathione-related antioxidant defenses in Pacific oysters, *Crassostrea gigas*. Mar. Environ. Res..

[20] Regoli F., Giuliani M.E. (2014). Oxidative pathways of chemical toxicity and oxidative stress biomarkers in marine organisms. Mar. Environ. Res..

[21] Sasaki S., Yokozawa T., Cho E.J., Oowada S., Kim M. (2006). Protective role of γ-aminobutyric acid against chronic renal failure in rats. J. Pharm. Pharmacol..

[22] Fathi M., Saeedyan S., Kaoosi M. (2023). Gamma-amino butyric acid (GABA) supplementation alleviates dexamethasone treatment-induced oxidative stress and inflammation response in broiler chickens. Stress.

[23] Hata T., Rehman F., Hori T., Nguyen J.H. (2019). GABA, γ-Aminobutyric Acid, Protects Against Severe Liver Injury. J. Surg. Res..

[24] Polenzani L., Woodward R., Miledi R. (1991). Expression of mammalian gamma-aminobutyric acid receptors with distinct pharmacology in Xenopus oocytes. Proc. Natl. Acad. Sci. USA.

[25] Kochiyama T., Li X., Nakayama H., Kage M., Yamane Y., Takamori K., Iwabuchi K., Inada E. (2019). Effect of propofol on the production of inflammatory cytokines by human polarized macrophages. Mediat. Inflamm..

[26] Tang X., Yu R., Zhou Q., Jiang S., Le G. (2018). Protective effects of γ-aminobutyric acid against H_2_O_2_-induced oxidative stress in RIN-m5F pancreatic cells. Nutr. Metab..

[27] Katila N., Bhurtel S., Park P., Choi D. (2021). Metformin attenuates rotenone-induced oxidative stress and mitochondrial damage via the AKT/Nrf2 pathway. Neurochem. Int..

[28] Oliveira M.R.d., Ferreira G.C., Schuck P.F., Dal Bosco S.M. (2015). Role for the PI3K/Akt/Nrf2 signaling pathway in the protective effects of carnosic acid against methylglyoxal-induced neurotoxicity in SH-SY5Y neuroblastoma cells. Chem.-Biol. Interact..

[29] Purwana I., Zheng J., Li X., Deurloo M., Son D.O., Zhang Z., Liang C., Shen E., Tadkase A., Feng Z.-P. (2014). GABA Promotes Human β-Cell Proliferation and Modulates Glucose Homeostasis. Diabetes.

[30] Zhang C., He J., Wang X., Su R., Huang Q., Qiao F., Qin C., Qin J., Chen L. (2022). Dietary gamma-aminobutyric acid (GABA) improves non-specific immunity and alleviates lipopolysaccharide (LPS)-induced immune overresponse in juvenile Chinese mitten crab (*Eriocheir sinensis*). Fish Shellfish Immunol..

[31] Bae J., Hamidoghli A., Farris N.W., Olowe O.S., Choi W., Lee S., Won S., Ohh M., Lee S., Bai S.C. (2022). Dietary γ-Aminobutyric Acid (GABA) Promotes Growth and Resistance to Vibrio alginolyticus in Whiteleg Shrimp *Litopenaeus vannamei*. Aquac. Nutr..

[32] Li J., Jiang M., Han Q., Peng R., Jiang X. (2020). Effects of γ-aminobutyric acid supplementation on the growth performance, serum biochemical indices and antioxidant status of pharaoh cuttlefish, *Sepia pharaonis*. Aquac. Nutr..

[33] MartínezGarcía M.F., Ruesink J.L., GrijalvaChon J.M., Lodeiros C., ArreolaLizárraga J.A., de la ReVega E., VarelaRomero A., ChávezVillalba J. (2022). Socioecological factors related to aquaculture introductions and production of Pacific oysters (*Crassostrea gigas*) worldwide. Rev. Aquac..

[34] Siboni N., King W.L., Williams N.L.R., Scanes E., Giardina M., Green T.J., Ostrowski M., O’Connor W., Dove M., Labbate M. (2024). Increased abundance of potentially pathogenic Vibrio and a marine heatwave co-occur with a Pacific oyster summer mortality event. Aquaculture.

[35] Park M.S., Jo P.G., Choi Y.K., An K.W., Choi C.Y. (2009). Characterization and mRNA expression of Mn-SOD and physiological responses to stresses in the Pacific oyster *Crassostrea gigas*. Mar. Biol. Res..

[36] Elia A.C., Burioli E., Magara G., Pastorino P., Caldaroni B., Menconi V., Dörr A.J.M., Colombero G., Abete M.C., Prearo M. (2020). Oxidative stress ecology on Pacific oyster *Crassostrea gigas* from lagoon and offshore Italian sites. Sci. Total Environ..

[37] Liu Z., Li M., Yi Q., Wang L., Song L. (2018). The neuroendocrine-immune regulation in response to environmental stress in marine bivalves. Front. Physiol..

[38] Yu Y., Tian D., Ri S., Kim T., Ju K., Zhang J., Teng S., Zhang W., Shi W., Liu G. (2023). Gamma-aminobutyric acid (GABA) suppresses hemocyte phagocytosis by binding to GABA receptors and modulating corresponding downstream pathways in blood clam, *Tegillarca granosa*. Fish Shellfish Immunol..

[39] Horton R.W., Meldrum B.S. (1973). Seizures induced by allylglycine, 3-mercaptopropionic acid and 4-deoxypyridoxine in mice and photosensitive baboons, and different modes of inhibition of cerebral glutamic acid decarboxylase. Br. J. Pharmacol..

[40] Hirai H., Sootome H., Nakatsuru Y., Miyama K., Taguchi S., Tsujioka K., Ueno Y., Hatch H., Majumder P.K., Pan B.-S. (2010). MK-2206, an allosteric Akt inhibitor, enhances antitumor efficacy by standard chemotherapeutic agents or molecular targeted drugs in vitro and in vivo. Mol. Cancer Ther..

[41] Jin J., Li Y., Liu Y., Jordan A.A., McIntosh J., Vargas J., Che Y., Yao Y., Wang M. (2021). Bispecific CD19-CD20 and CD19-CD22 CAR-T Cells with Glycogen Synthase Kinase (GSK)-3β Inhibitor TWS119 Treatment Have Superior Therapeutic Effects on Mantle Cell Lymphoma. Blood.

[42] Wang W., Zhang T., Wang L., Xu J., Li M., Zhang A., Qiu L., Song L. (2016). A new non-phagocytic TLR6 with broad recognition ligands from Pacific oyster *Crassostrea gigas*. Dev. Comp. Immunol..

[43] Xing Z., Gao L., Liu R., Yang Q., Li Q., Wang L., Song L. (2023). The oxidative stress of the Pacific oyster *Crassostrea gigas* under high-temperature stress. Aquaculture.

[44] Lee M., Schwab C., McGeer P.L. (2011). Astrocytes are GABAergic cells that modulate microglial activity. Glia.

[45] Dong W., Liu Z., Qiu L., Wang W., Song X., Wang X., Li Y., Xin L., Wang L., Song L. (2017). The modulation role of serotonin in Pacific oyster *Crassostrea gigas* in response to air exposure. Fish Shellfish Immunol..

[46] Rahman M.S., Rahman M.S. (2021). Effects of elevated temperature on prooxidant-antioxidant homeostasis and redox status in the American oyster: Signaling pathways of cellular apoptosis during heat stress. Environ. Res..

[47] Ruenkoed S., Nontasan S., Phudkliang J., Phudinsai P., Pongtanalert P., Panprommin D., Mongkolwit K., Wangkahart E. (2023). Effect of dietary gamma aminobutyric acid (GABA) modulated the growth performance, immune and antioxidant capacity, digestive enzymes, intestinal histology and gene expression of Nile tilapia (*Oreochromis niloticus*). Fish Shellfish Immunol..

[48] Menon S.V., Kumar A., Middha S.K., Paital B., Mathur S., Johnson R., Kademan A., Usha T., Hemavathi K.N., Dayal S. (2023). Water physicochemical factors and oxidative stress physiology in fish, a review. Front. Environ. Sci..

[49] Zhao J., Zhao B., Kong N., Li F., Liu J., Wang L., Song L. (2023). Increased abundances of potential pathogenic bacteria and expressions of inflammatory cytokines in the intestine of oyster *Crassostrea gigas* after high temperature stress. Dev. Comp. Immunol..

[50] Meistertzheim A.L., Tanguy A., Moraga D., Thébault M.T. (2007). Identification of differentially expressed genes of the Pacific oyster *Crassostrea gigas* exposed to prolonged thermal stress. Febs J..

[51] Chen C., Li P., Wang W., Li Z. (2022). Response of growth performance, serum biochemical parameters, antioxidant capacity, and digestive enzyme activity to different feeding strategies in common carp (*Cyprinus carpio*) under high-temperature stress. Aquaculture.

[52] Li A., Li L., Song K., Wang W., Zhang G. (2017). Temperature, energy metabolism, and adaptive divergence in two oyster subspecies. Ecol. Evol..

[53] Ding F., Li A., Cong R., Wang X., Wang W., Que H., Zhang G., Li L. (2020). The phenotypic and the genetic response to the extreme high temperature provides new insight into thermal tolerance for the pacific oyster *Crassostrea gigas*. Front. Mar. Sci..

[54] Lu Y., Wu Z., Song Z., Xiao P., Liu Y., Zhang P., You F. (2016). Insight into the heat resistance of fish via blood: Effects of heat stress on metabolism, oxidative stress and antioxidant response of olive flounder *Paralichthys olivaceus* and turbot *Scophthalmus maximus*. Fish Shellfish Immunol..

[55] Sun J., Zhao L., Liao L., Tang X., Cui C., Liu Q., He K., Ma J., Jin L., Yan T. (2020). Interactive effect of thermal and hypoxia on largemouth bass (*Micropterus salmoides*) gill and liver: Aggravation of oxidative stress, inhibition of immunity and promotion of cell apoptosis. Fish Shellfish Immunol..

[56] Hogg D.W., Hawrysh P.J., Buck L.T. (2014). Environmental remodelling of GABAergic and glutamatergic neurotransmission: Rise of the anoxia-tolerant turtle brain. J. Therm. Biol..

[57] Nilsson G.E. (1992). Evidence for a role of gaba in metabolic depression during anoxia in crucian carp (*Carassius carassius*). J. Exp. Biol..

[58] Li M., Wang L., Qiu L., Wang W., Xin L., Xu J., Wang H., Song L. (2016). A glutamic acid decarboxylase (*Cg*GAD) highly expressed in hemocytes of Pacific oyster *Crassostrea gigas*. Dev. Comp. Immunol..

[59] Liang Y., Wu Y., Li J., Peng R., Jiang M., Jiang X., Chen S., Lin J. (2022). Effects of ammonia toxicity on the histopathology, detoxification, oxidative stress, and immune response of the cuttlefish *Sepia pharaonis* and the mitigation of γ-aminobutyric acid. Ecotoxicol. Environ. Saf..

[60] Guan X., Shi W., Zha S., Rong J., Su W., Liu G. (2018). Neurotoxic impact of acute TiO_2_ nanoparticle exposure on a benthic marine bivalve mollusk, *Tegillarca granosa*. Aquat. Toxicol..

[61] Haider F., Falfushynska H.I., Timm S., Sokolova I.M. (2020). Effects of hypoxia and reoxygenation on intermediary metabolite homeostasis of marine bivalves *Mytilus edulis* and *Crassostrea gigas*. Comp. Biochem. Physiol. Part A Mol. Integr. Physiol..

[62] Lauder J.M. (1993). Neurotransmitters as growth regulatory signals: Role of receptors and second messengers. Trends Neurosci..

[63] Hori T., Uemoto S., Walden L.B., Chen F., Baine A.T., Hata T., Nguyen J.H. (2013). Pretreatment of small-for-size grafts in vivo by γ-aminobutyric acid receptor regulation against oxidative stress-induced injury in rat split orthotopic liver transplantation. Int. J. Hepatol..

[64] Richmond J.E., Murphy A.D., Lukowiak K., Bulloch A.G.M. (1994). GABA regulates the buccal motor output of *Helisoma* by two pharmacologically distinct actions. J. Comp. Physiol. A.

[65] Hutton M.L., Harvey R.J., Earley F.G.P., Barnard E.A., Darlison M.G. (1993). A novel invertebrate GABAA receptor-like polypeptide Sequence and pattern of gene expression. FEBS Lett..

[66] Jiang W., Fang J., Rastrick S.P.S., Samuelsen O.B., Liang B., Mao Y., Strand Ø., Fang J., Jiang Z. (2023). CO_2_-Induced ocean acidification alters the burrowing behavior of Manila clam *Ruditapes philippinarum* by Reversing GABAA receptor function. Environ. Sci. Technol..

[67] Johnston G.A.R. (2013). Advantages of an antagonist: Bicuculline and other GABA antagonists. Br. J. Pharmacol..

[68] Deng Z., Li D., Wang L., Lan J., Wang J., Ma Y. (2024). Activation of GABABR attenuates intestinal inflammation by reducing oxidative stress through modulating the TLR4/MyD88/NLRP3 pathway and gut microbiota abundance. Antioxidants.

[69] Khan M.Z., Khan A., Chen W., Chai W., Wang C. (2024). Advancements in genetic biomarkers and exogenous antioxidant supplementation for safeguarding mammalian cells against heat-induced oxidative stress and apoptosis. Antioxidants.

[70] Lin Y., Li X., Chen X., Chen J., Jin X., Sun J., Niu X., Kong Y., Li M., Wang G. (2024). γ-aminobutyric acid effectively modulate growth performance, physiological response of Largemouth bass (*Micropterus salmoides*) under combined stress of flow velocity and density. Aquac. Nutr..

[71] Yan Z., Liu B., Liu J., Guo Z., Kou Y., Lu W., Sun J., Li Y. (2024). Enhancing resilience to chronic ammonia stress in crucian carp (*Carassius carassius*) through dietary gamma-aminobutyric acid (GABA) supplementation: Effects on growth performance, immune function, hepatotoxicity, and apoptosis. Aquac. Rep..

[72] Saha S., Buttari B., Panieri E., Profumo E., Saso L. (2020). An overview of Nrf2 signaling pathway and its role in inflammation. Molecules.

[73] Soltani N., Qiu H., Aleksic M., Glinka Y., Zhao F., Liu R., Li Y., Zhang N., Chakrabarti R., Ng T. (2011). GABA exerts protective and regenerative effects on islet beta cells and reverses diabetes. Proc. Natl. Acad. Sci. USA.

[74] Gardner L.B., Hori T., Chen F., Baine A.T., Hata T., Uemoto S., Nguyen J.H. (2012). Effect of specific activation of γ-aminobutyric acid receptor in vivo on oxidative stress-induced damage after extended hepatectomy. Hepatol. Res..

[75] Salazar M., Rojo A.I., Velasco D., de Sagarra R.M., Cuadrado A. (2006). Glycogen synthase kinase-3beta inhibits the xenobiotic and antioxidant cell response by direct phosphorylation and nuclear exclusion of the transcription factor Nrf2. J. Biol. Chem..

[76] Chowdhry S., Zhang Y., McMahon M., Sutherland C., Cuadrado A., Hayes J.D. (2013). Nrf2 is controlled by two distinct β-TrCP recognition motifs in its Neh6 domain, one of which can be modulated by GSK-3 activity. Oncogene.

[77] Hou L., Qiao X., Li Y., Jin Y., Liu R., Wang S., Zhou K., Wang L., Song L. (2022). A RAC-alpha serine/threonine-protein kinase (*Cg*AKT1) involved in the synthesis of *Cg*IFNLP in oyster *Crassostrea gigas*. Fish Shellfish Immunol..

[78] Sun J., Gao L., Huang S., Wang L., Yang W., Zhang T., Jin Y., Song L. (2021). CLec-TM1–ERK–GSK3β pathway regulates Vibrio splendidus–induced IL-17 production in oyster. J. Immunol..

